# Biomechanical Insights in Ancient Greek Combat Sports: A Static Analysis of Selected Pottery Depictions

**DOI:** 10.3390/sports12120317

**Published:** 2024-11-26

**Authors:** Andreas Bourantanis, Nikitas Nomikos, Weijie Wang

**Affiliations:** 1 Department of Orthopaedic and Trauma Surgery, School of Medicine, University of Dundee, Dundee DD1 9SY, UK; abourantanis@dundee.ac.uk; 2 Department of Health Sciences and Sports, Medical School, National and Kapodistrian University of Athens, Mikras Asias 75, 11527 Athens, Greece; niknomikos@med.uoa.gr

**Keywords:** Pankration, ancient athletics, combat sports, sports biomechanics, static analysis

## Abstract

Background: Though ancient Greece preserves many pictures of combat sports, there is limited research in terms of biomechanical analysis of their sports. This research aimed to investigate the Pankration postures of ancient Greek athletics, expecting to bridge the gap between historical sports practices and contemporary biomechanical applications. Methods: This study employed computer vision (OpenPose) to analyze two images, one as readiness and another as kicking postures, from ancient Greek Pankration by constructing a static multi-segmental model. Using Newton’s Laws, the models simulated the postures as presented in historical depictions, estimated joint forces and moments, and calculated weight distribution and ground reaction forces for these postures. Results: For the readiness posture, it was found that the right hind leg experienced significant forces, with the highest moment at the knee joint, while the ankle and hip joints showed similar slightly lower moments. The front leg encountered lower forces and moments. For the kick posture, the supporting leg experienced the highest moment at the knee, while the kicking leg showed minimal moments at the ankle, knee, and hip. Conclusions: The static analysis provided quantitative estimates of joint forces and moments in the depicted Pankration postures, suggesting that these postures were biomechanically effective for their intended functions in combat. While the analysis cannot confirm whether ancient athletes deliberately applied biomechanical principles, the results highlight the potential of biomechanical modeling to enhance our understanding of ancient sports practices. The research demonstrates the possible benefits of integrating static analysis with historical elements to study the physical demands and techniques of ancient combat sports.

## 1. Introduction

Biomechanics, as a scientific discipline, originated from a long-standing interest in human anatomy and motion principles. Its earliest roots can be traced back to Egyptian papyri around 1700–1600 BC [1]. Pioneers like Hippocrates and Galen of Pergamon furthered this curiosity, laying the groundwork for biomechanics [2]. This foundational knowledge has been of great importance across various fields, particularly in analyzing biomechanical aspects of ancient sports practices, such as those originating in Greece [3].

The ancient Greek sports have captivated researchers, who have used historical and archaeological evidence to reconstruct athletic techniques, including the long jump and discus throw [4]. These reconstructions have primarily focused on kinematics (the study of movement) without considering the forces behind it. Despite technological advancements, a detailed reconstruction of these ancient techniques remains lacking, largely due to the fragmentary nature of the available sources.

Recently, researchers have analyzed the Centre of Mass (CoM) in ancient sports, recognizing its critical role in biomechanical analysis. Controlling the CoM is essential for maintaining balance and reducing injury risk [5]. Some studies have investigated how posture maintenance relates to sports performance and injury prevention [6] and emphasized the importance of balance training for enhancing proprioceptive abilities, crucial for spatial awareness in sports [7]. This balance training has helped athletes transition effectively between static and dynamic states, providing a competitive edge [8].

In sports, mastering control of CoM and balance is of heightened significance. Athletes adjust their CoM for stability and to execute powerful movements. Disrupting an opponent’s balance is a strategic element, which underscores the mastery of CoM manipulation [9]. This intricate relationship between balance and movement naturally leads us to consider the underlying mechanical principles that govern athletic postures.

Statics, a branch of mechanics that deals with bodies at rest or in uniform motion (constant velocity), is essential for analyzing the forces and moments acting on an athlete’s body when maintaining or transitioning between postures. It involves studying systems in equilibrium, where the sum of all forces and the sum of all moments acting upon the body are zero. Researchers can understand how athletes maintain balance and how different postures affect the biomechanical parameters in the joints. Statics have been used as a tool to estimate the relationship between an athlete’s postures and biomechanical parameters in the joints [10]. To understand optimal postures for performance, researchers in sports biomechanics have increasingly turned to static optimization [11], which involves adjusting variables within a static model to find the most efficient posture for a given task.

Therefore, statics is used as a tool to explore the relationship between an athlete’s postures and biomechanical parameters in the joints [10]. To understand optimal postures for performance, researchers in sports biomechanics have increasingly turned to static optimization [11].

Static optimization and optimal control are crucial for enhancing athletic performance, designing targeted training programs, and improving rehabilitation protocols [12]. A fully biomechanical analysis of athletes also requires investigating the forces and moments acting on the legs, along with the ground reaction force (GRF). The GRF is reactive to the exertions of the body and is key to understanding how athletes maintain equilibrium, orchestrate movement, and generate power in both static postures and dynamic activities [13]. Despite these advancements, the static analysis of the postures of ancient athletes remains a relatively unexplored area [4,5]. Though current research has enhanced our understanding of movement kinematics [4,14], the static aspects, such as the specific postures and alignments used by ancient athletes, lack a comprehensive study. Given the significance of posture in modern sports for performance enhancement, exploring the posture of ancient athletics could provide a valuable reference to modern sports [15,16]. Static analysis could reveal how ancient athletes prepared for and executed their maneuvers, offering deeper insights into their training techniques. Hence, this study aimed to investigate the postures of ancient Greek athletes in terms of biomechanical parameters, especially the joint forces and moments produced while they maintained specific stances. The primary objective of the present research was to build a static multi-segmental model utilizing computer vision techniques and investigate the biomechanical parameters in ancient Greek combat postures. Ultimately, this study aimed to highlight the need for a comprehensive biomechanical analysis of Pankration postures. This need was addressed by taking the first step through a fundamental static analysis of two selected Pankration postures. By reevaluating ancient knowledge, particularly in sports with roots in antiquity, it should be attempted to reconstruct these techniques without introducing modern influences. This study is justified as it aims to assist in examining aspects (statics) of the ancient combat sports practices as it focuses on examining their biomechanics, leveraging only preserved artifacts of ancient Greece.

## 2. Methods

Recent advancements in machine learning provide tools that can potentially support biomechanical analysis [17,18,19].

For the present research, OpenPose was used. OpenPose is a multi-stage Convolutional Neural Network (CNN) that predicts 2D confidence maps and part affinity fields for accurate human pose estimation [20]. OpenPose was selected for its capacity to process two-dimensional images and generate accurate keypoint data with minimal human intervention. Previous studies have validated its effectiveness in various sports biomechanics applications [21,22,23]. OpenPose was applied to analyze ancient Greek Pankration depictions, extracting keypoint data. Using these data, the body segments were constructed to form a static multi-segmental model, which enabled the quantification of visual information from the depictions.

**Figure 1:**
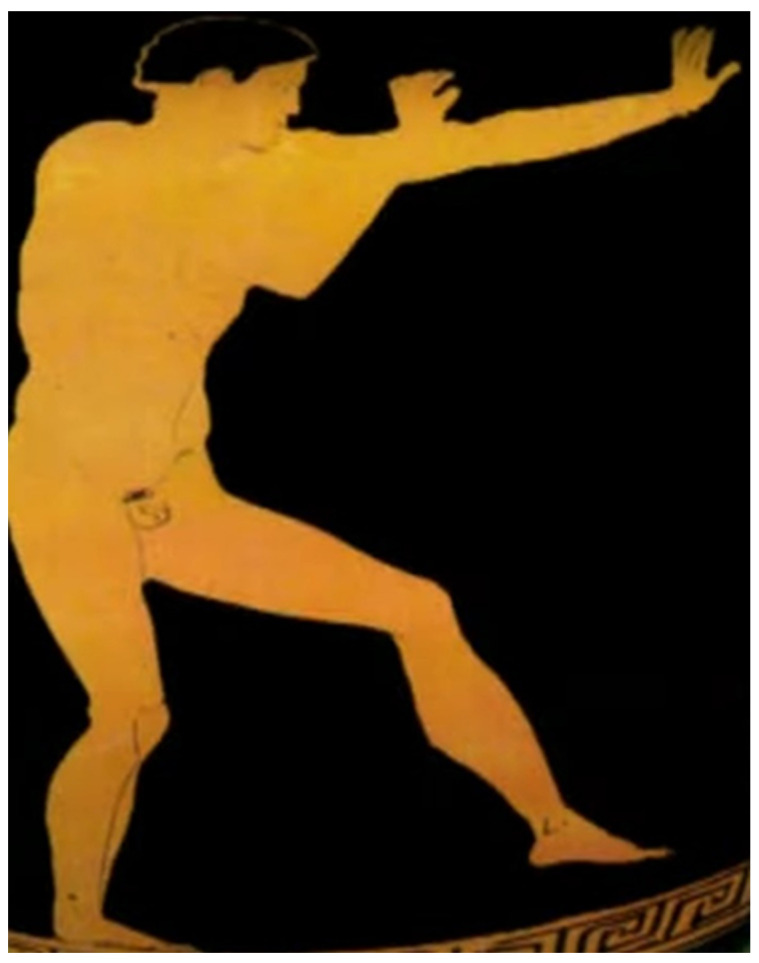
A typical readiness posture of ancient Greek athletes in combat. The image depicts an athlete engaged in the ancient Greek sport of Pankration, as illustrated on a red-figure amphora housed in the Staatliche Antikensammlungen Museum in Munich, Germany [24].

**Figure 2:**
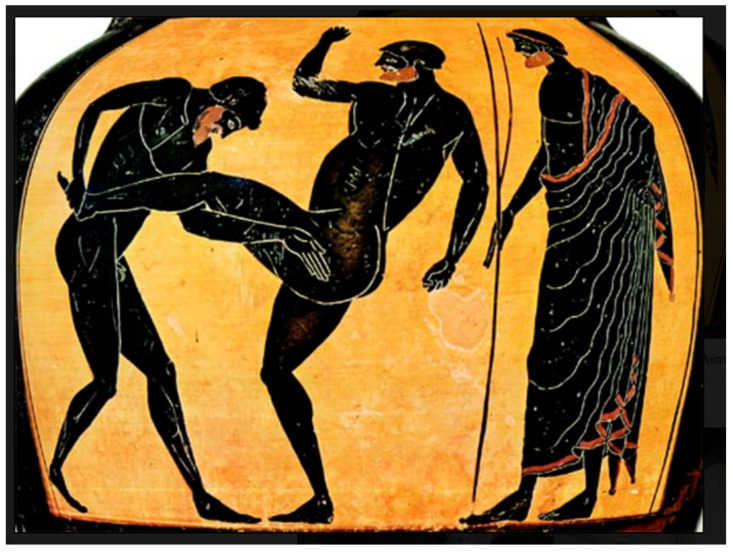
The final posture presents an athlete who receives a kick, possibly aimed at the opponent’s abdomen area. The present illustrates a scene of a Pankration match depicted on a black-figure amphora from the classical period (5th century BCE) [24].

In this investigation, two posture representations were used (Figure 1 and Figure 2), each exemplifying a critical phase of ancient Pankration. After the selection of these depictions, OpenPose was employed to analyze the images.

**Figure 3:**
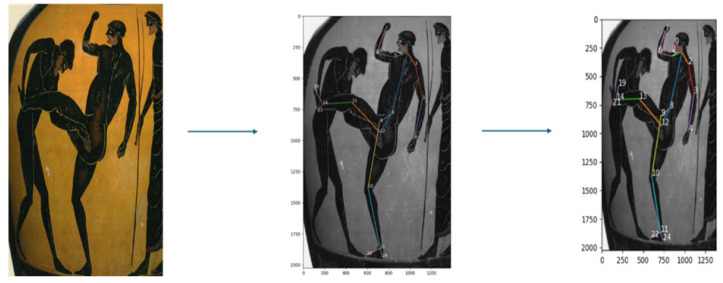
Modified foot position emphasizing the heel strike. The altered foot orientation is consistent with ancient textual descriptions of defensive maneuvers, as exemplified by the attacker–defender interaction depicted in Figure 2.

As both figures have been derived from photographs of the original artifacts, which are vases, the inherent curvature in Figure 1 introduces distortions that could mislead the viewer into interpreting the image as having one foot off the ground. This issue arises because the two feet are not aligned in the same line. However, based on differential geometry, which accounts for how curvature affects visual perception, the most probable scenario is that both feet are on the ground. In sports biomechanics, it is necessary that both feet are grounded for balance. Along with the literature that describes the depicted posture, we have chosen the most probable scenario where both feet were in contact with the ground [25,26]. It is also documented that the vase curvature affects the visualization of depth [27], making it seem as though the legs are not on the ground. For the purposes of this research, we considered the feet to be separated by a gap, visualized in depth, despite the depiction being in the sagittal plane. This interpretation is favored as it is supported by both the ancient and modern literature on athletic postures and reinforced by mathematical and biomechanical perspectives [24,28,29,30]. In accordance with ancient textual references to the heel strike [31], the orientation of the foot in the second image was adjusted such that the heel constitutes the leading edge. This modification visually represents the defensive action of deflecting the attacker’s assault, as depicted in Figure 2. The modification is visible in Figure 3.

### 2.1. Segmental and Total CoM

The determination of the centre of mass (CoM of each body segment and the overall CoG was achieved through a Python script. This code was developed to process keypoint data extracted from OpenPose, serving as a tool for easier interaction with OpenPose–generated files.

The script’s functionality centered on the computation of the CoM within a two-dimensional Cartesian coordinate system. This computation relies on the positional data of keypoints, as detected by OpenPose. The underlying mathematical model for the CoM is encapsulated in the following formula:

(1) $$\mathrm{CoM}x=\frac{\sum m_{i}\cdot x_{ic}}{M}$$

(2) $$\mathrm{CoM}y=\frac{\sum m_{i}\cdot y_{ic}}{M}$$

Equation (1) calculates the x-coordinate of the CoM as the sum of the products of each body segment’s mass, $m_{i}$, and its x-coordinate, $x_{ic}$, divided by the total mass M. The term $m_{i}$ represents the mass associated with the i-th keypoint, and $x_{ic}$ denotes the x-coordinate of the center of mass for each segment. Similarly, the second Equation (2) calculates the y-coordinate of the CoM using the same principle, where $y_{ic}$ is the y-coordinate of the center of mass for each segment. [32,33,34].

### 2.2. Static Multi-Segmental Model of the Lower Extremity

In this study, a static multi-segmental analysis was applied to the lower extremity, structured progressively from the foot to the pelvis. This approach adopted a static equilibrium perspective, concentrating exclusively on the gravitational forces and moments acting upon each posture, thus overlooking other relevant factors such as the dynamic forces and kinematic properties of movement.

The underlying assumption for each segmental analysis was the state of static equilibrium, which, in mechanical terms, is defined by the conditions that the sum of forces in the horizontal (ΣFx=0) and vertical (ΣFy=0) directions, as well as the sum of moments about any position (ΣM=0), must be zero. These conditions are derived from the fundamental principles of statics in rigid body mechanics [35].

Given the complexity inherent in analyzing the kinetics of a large free body, a strategy of decomposition into smaller sub-free bodies was employed [36].

**Figure 4:**
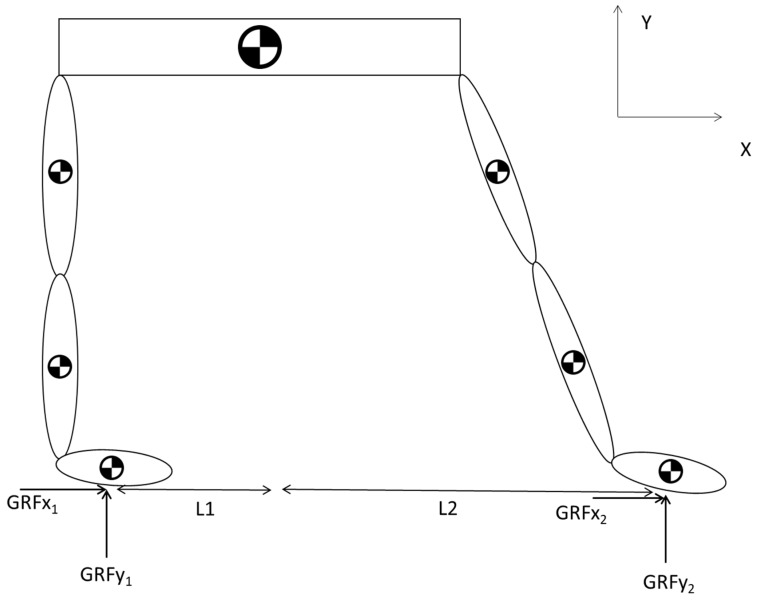
Biomechanical model of lower limbs in the sagittal plane, used for calculating ground reaction forces. The model was used to estimate the ground reaction forces (GRFs). The model consisted of seven segments: two thighs, two shanks, two feet, and the pelvis (represented by the horizontal rectangle connecting the limbs).

The lower extremity was divided into its constituent segments for detailed analysis. These segments typically include the thigh (upper leg), shank (lower leg), and foot. The division is based on anatomical landmarks and was crucial for isolating the forces and moments acting on each segment. A free-body diagram was constructed for each segment of the lower extremity, as shown in Figure 4. The analysis was initiated by ascertaining the Ground Reaction Force (GRF), which refers to the forces exerted at the base of the foot in response to gravitational interaction. Since the modeling was conducted in the sagittal plane (2D), the focus was solely on the vertical and horizontal forces. The horizontal force, which is parallel to the ground, is affected by the friction, which is defined as:

(3) $$GRFx_{1}=Co\times GRFy_{1}$$

Wherein ‘*Co*’ denotes the coefficient of friction, assigned a value of 0.3, a characteristic extrapolated from the literature, which informed us about the execution of sporting events on surfaces layered with sand [37]. $GRF_{x1}$ and $GRF_{y1}$ are the components of the force along the corresponding axes, $x_{1}$ and $y_{1}$, and they can be adapted according to the situation, representing the hind and front legs, respectively. The Ground Reaction Forces (GRF) for each leg can be calculated by applying the principles outlined in the free body diagram provided in Figure 4.

Therefore, $GRFx_{2}$ can be calculated as follows:

(4) $$GRFx_{2}=-GRFx_{1}$$

The next step was to ascertain the individual vertical ground reaction forces (GRFy) acting on each foot; the equilibrium equation was employed for the system in conjunction with the horizontal ground reaction forces [38,39].

Using Newton Laws for the whole system, the static equations include forces and moment components as follows:

(5) $$\Sigma F_{x}=GRF_{x1}+GRF_{x2}=0$$

(6) $$\Sigma F_{y}=GRF_{y1}+GRF_{y2}-P_{total}=0$$

(7) $$\Sigma M=P_{total}\times L2-GRF_{y1}\times \left(L1+L2\right)=0$$

To explain the graph and equations, we need to consider a static equilibrium analysis of a system, particularly focusing on the vertical ground reaction forces (GRFs) on the legs. This involves summing the forces and moments to ensure equilibrium. Equation (5) states that the sum of all horizontal forces acting on the system is zero. In this context, *GRFx*_1_ represents the horizontal ground reaction force on the first leg, and *GRFx*_2_ represents the horizontal ground reaction force on the second leg. For the system to be in horizontal equilibrium, the horizontal ground reaction forces must be equal in magnitude but opposite in direction, thereby cancelling each other out.

Equation (6) states that the sum of all vertical forces acting on the system must be zero. Here, $GRFy_{1}$ is the vertical ground reaction force on the first leg, $GRFy_{2}$ represents the vertical ground reaction force on the second leg, and $P_{total}$ is the total vertical load applied to the system, such as the weight of the person. For vertical equilibrium, the total vertical ground reaction forces must equal the total applied load. Equation (7) represents the moment equilibrium about the base of one leg. In this case, $P_{total}$ is the total vertical load, i.e., the body weight. L1 the horizontal distance from the base of the first leg to the point where the force of the CoM acts and L2  is the horizontal distance from the base of the second leg to the coordinates of the CoM. For moment equilibrium about the base of one leg, the total moment due to the applied load must equal the total moment due to the vertical ground reaction force on that leg. The product $P_{total}\;\times \;L2$ provides the moment caused by the total load about the base of the first leg.

**Figure 5:**
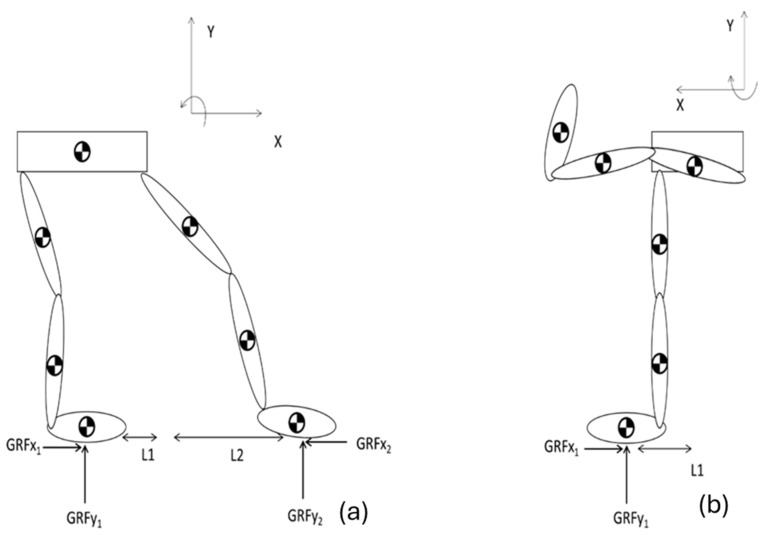
Biomechanical model of the lower limbs in the sagittal plane, adjusted for calculating ground reaction forces across different postures. The model, consisting of seven segments—two thighs, two shanks, two feet, and the pelvis (represented by the horizontal rectangle connecting the limbs)—was used to estimate ground reaction forces (GRF) while accounting for variations in posture, joint angles, and load distribution. The left free body diagram (**a**) represents the initial posture found in Figure 1 while the right (**b**) corresponds to Figure 2. It is important to note that, due to the kick facing the opposite direction compared to the readiness posture, the reference system has been adjusted to align with the depictions. Specifically, while the negative sign in the readiness posture indicates clockwise moments, this convention is reversed in the second depiction, where the negative sign now represents a counterclockwise moment.

The general model, originally designed for ground reaction force (GRF) analysis (Figure 4), was modified to incorporate the specific attributes and circumstances depicted in the two images analyzed in the present research (Figure 5). Through the integration of these modifications, the model has been refined to account for variations in posture, joint angles, and joint load distribution, thus enabling a more thorough analysis of the forces and moments acting on the body under different conditions. Additionally, this refinement presents the model’s versatility and its potential applicability to real-world biomechanical scenarios. Therefore, having derived the necessary values, the analysis was initiated at the first segment, i.e., the foot.

**Figure 6:**
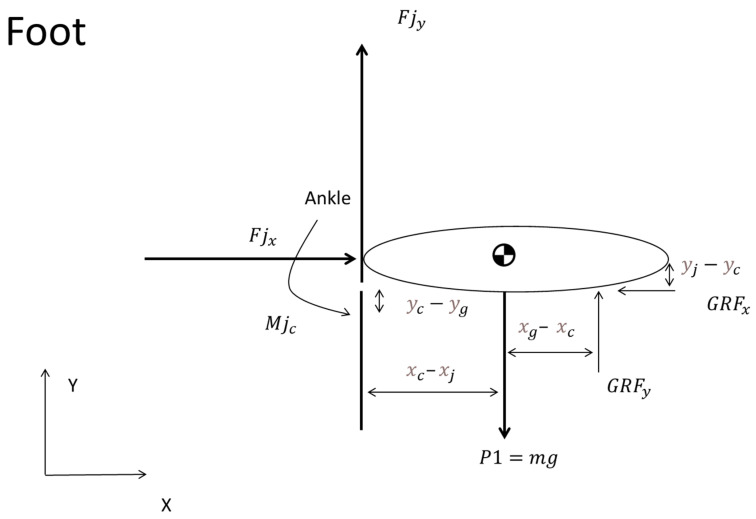
Diagram illustrating the equilibrium of forces on the foot. The figure is a free body diagram that presents the lever arms in vector form, along with the forces and moments acting on the respective segment, i.e., the foot.

The foot, as illustrated in Figure 6, served as a foundational tool in this analytical process. It visually represents all the forces and moments acting on the foot, which is treated as a rigid body.

For the foot segment, the equilibrium conditions are expressed by the following Equations (8)–(10):

(8) $$\Sigma F_{a}=F_{jx}-GRF_{x}$$

(9) $$\Sigma F_{a}=F_{jy}+GRF_{y}-P1$$

(10) $$M_{jc}-F_{jy}\times \left(X_{c}-X_{j}\right)-F_{jx}\times \left(y_{j}-y_{c}\right)+F_{gy}\times \left(x_{g}-X_{c}\right)+F_{gx}\times \left(y_{c}-y_{g}\right)=0$$

The forces $F_{jx}$ and $F_{jy}$ represent the joint forces in the horizontal and vertical directions, respectively, (8) and (9). In Equation (10), $M_{jc}$ denotes the joint moment. Concurrently, $Fg_{x}$ and $Fg_{y}\;$ correspond to the ground reaction forces (GRFs) in the horizontal and vertical planes. P1 is the foot weight, computed as the product of the mass of the foot and the gravitational acceleration [36].

The coordinates ($x_{j},\;y_{j}$) mark the ankle joint’s location, acting as a crucial point for the application of joint forces, whereas ($x_{c}$, $y_{c}$), indicate the foot’s center of mass. In the same manner, ($x_{g},\;y_{g}$) pinpoint the contact areas between the foot and the ground, where the GRFs are exerted (Figure 6).

**Figure 7:**
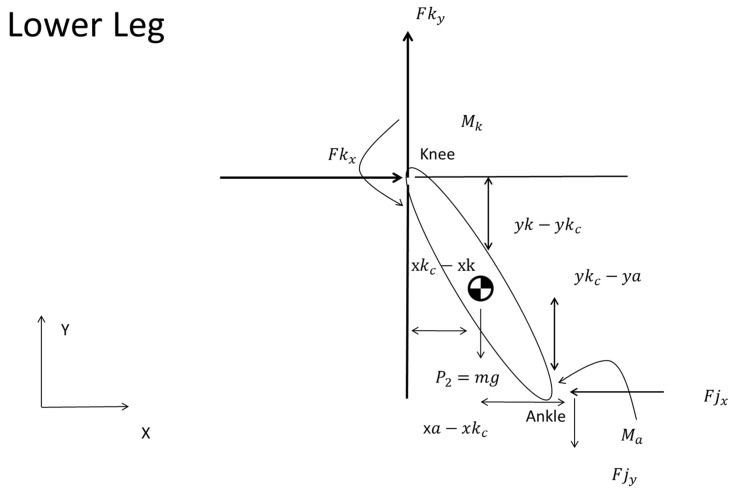
Representation of lower leg forces from ankle to knee, showing equilibrium conditions.

Advancing to the lower leg (ankle to knee joint) (Figure 7), the model imposed similar equilibrium conditions, now referencing the forces and moments acting upon the ankle, articulated by Equations (11)–(13), as follows:

(11) $$\Sigma F_{k_{x}}=F_{kx}-F_{jx}$$

(12) $$\Sigma F_{k_{y}}=F_{ky}-F_{jy}-P2$$

(13) $$-M_{a}+M_{kc}-F_{kx}\times \left(y_{k}-y_{kc}\right)-F_{ky}\times \left(x_{kc}-x_{k}\right)-F_{jy}\times \left(x_{a}-x_{kc}\right)-F_{jx}\times \left(y_{kc}-y_{a}\right)=0$$

Starting with Equation (11), $F_{kx}$ represents the horizontal force at the knee joint and in Equation (12), $F_{ky}$ is the vertical component.

Within this framework, $M_{a}$ and $M_{kc}$ represent the moments at the ankle and knee, respectively, indicative of the rotational effects engendered by the forces acting on the segment (13).

In Equations (12) and (13), P2 is the gravitational force, i.e., the weight of the lower leg, and m is the mass of the shank segment. The coordinates ($x_{k},\;y_{k}$) and ($x_{a},\;y_{a}$) designate the positions of the knee and ankle joints in the plane. Furthermore, ($x_{kc},\;y_{kc}$) specified the CoM of the calf, as indicated by the literature (Figure 7).

The forces and moments exerted on one segment are invariably transferred to the adjacent segment. In the same manner, the method is extended to the femur. The following diagram describes the moments and forces acting on the femur, which is the next segment where forces and moments act from the preceding segment. Therefore, we applied

(14) $$\Sigma F_{x}=F_{hx}-F_{kx}=0$$

(15) $$\Sigma F_{y}=F_{hy}-F_{ky}-P3=0$$

(16) $$-M_{kc}+M_{hc}-F_{hx}\times \left(y_{h}{-y}_{hc}\right)-F_{hy}\times \left(x_{hc}-x_{h}\right)-F_{ky}\times \left(x_{k}-x_{hc}\right)-F_{kx}\times \left(y_{hc}-y_{k}\right)=0$$

Equation (14) establishes the balance of horizontal forces, denoting that the horizontal force component at the hip joint ($F_{hx}$) was counterbalanced by the horizontal force component at the knee joint ($F_{kx}$). Equation (15) demonstrates the vertical force equilibrium. The vertical component of the force at the hip joint ($F_{hy}$) minus the vertical component of the knee joint force ($F_{ky}$) and the force P3 sum to zero.

**Figure 8:**
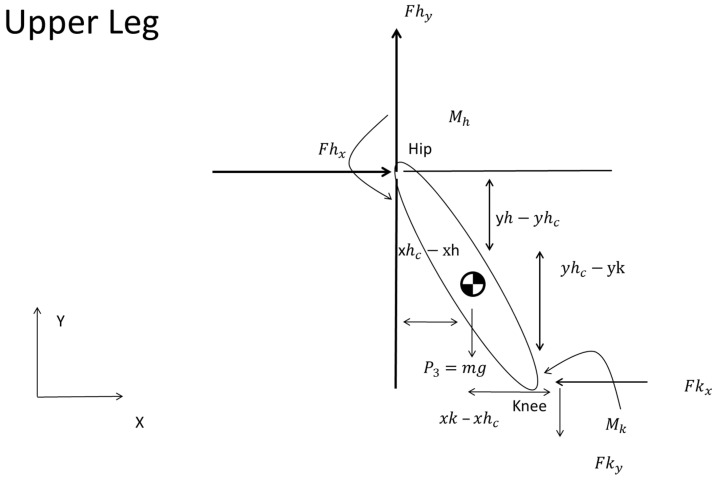
Analysis of the femur demonstrating force and moment equilibrium, as forces are transmitted from the knee to the hip joint.

In the same manner, Equation (16) defined the moment equilibrium about the hip joint. $M_{kc}$ and $M_{hc}$ are the moments at the knee and hip about the CoM of the femur (Figure 8). The coordinates $Y_{hc}$ and $X_{hc}$ specify the CoM of the femur. Meanwhile, $Y_{h}$ and $X_{h}$ pinpoint the locations of the hip and knee (Figure 8).

### 2.3. Anthropometric Parameters of a Hypothetical Male Athlete Model

The anthropometric model used was based on an assumed male athlete with a stature of 185 cm and a total body mass of 100 kg. This model assumed standard proportions for body segments, which were used for estimating parameters such as CoM, joint moments, and force distributions. The height and mass values were chosen to reflect the characteristics of a typical male athlete, providing a representative baseline for biomechanical analysis.

The model was divided into anatomical segments with the following specified lengths (Table 1), while the literature provides the following relationships for each segment (Table 2) [36], as follows:

## 3. Results

**Figure 9:**
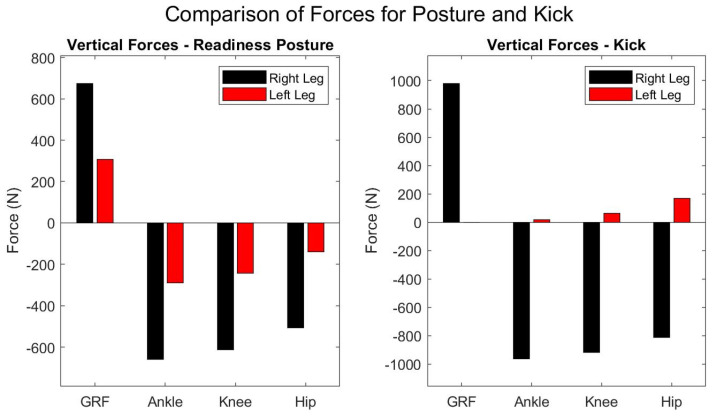
Vertical forces on lower extremity joints during “readiness” static posture and kick posture highlight differences in force distribution between the right and left legs. Since the vertical forces were calculated, it is clear that the reactions will have opposite signs. As the weight of each segment is subtracted, the vertical force progressively decreases from the foot up to the hip.

The current analysis provided insights into the forces experienced by the athletes and the forces and the moments influencing the key joints in the lower limbs. By examining the forces and moments acting on the hip, knee, and ankle, this study tried to highlight the mechanical loads these joints endure. Additionally, the moments around these joints provided crucial information on how the body maintains stability, as demonstrated by the results presented in Figure 9.

The right leg, serving as the hind leg, experienced significant forces. The horizontal ground reaction force (GRFX1) was calculated at 202.35 N. Since the horizontal force was defined as the product of friction, it can vary depending on the surface. However, it still provided a reasonable estimate of the magnitude of the force applied horizontally.

The vertical ground reaction force (GRFY1) at 674.5038 N reflects the substantial load the right leg bears, corresponding to body weight and the additional biomechanical demands of maintaining posture and readiness for movement. This is further evidenced by the substantial vertical forces at the ankle, knee, and hip joints, indicative of the right leg’s involvement in power generation and absorption of impacts (Figure 9), as well as the horizontal forces that were calculated, which had a magnitude of 202.35 N.

The hind leg exhibited notable torques across all joints. The ankle joint moment was estimated at −33.2249 Newton meters (Nm). The negative sign indicates the activation of the gastrocnemius muscle prompting plantar flexion. The knee joint, with a moment of 49.7192 Nm, was identified as bearing the highest load among the assessed joints, which indicates the possible role in generating propulsive forces for strikes and in absorbing impacts during various combat movements [33]. The positive sign here refers to the use of the extensor muscles and their possible contribution to the posture’s dynamics as indicated by the static analysis [36]. The hip joint moment was registered at −32.3679 Nm with a magnitude almost identical to that of the ankle joint, necessitating a [36] strong activation of the hip extensors (Figure 9) [36].

In contrast, the front (left) leg encounters a mirrored horizontal ground reaction force of −202.3511 N and a reduced vertical force of 306.4962 N, suggesting lesser load-bearing.

**Figure 10:**
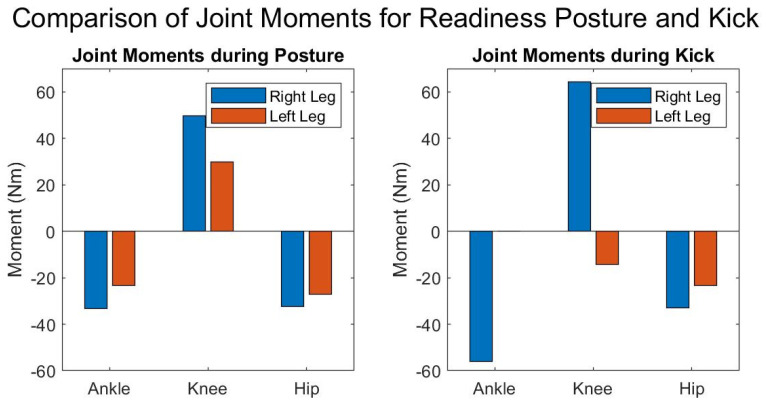
Joint moments at the ankle, knee, and hip for “readiness” posture (**left**) and kick posture (**right**). Positive moments indicate counterclockwise rotation, and negative moments indicate clockwise rotation.

The ankle joint experienced a joint moment e of −23.3398 Nm. The knee joint moment, at 29.8407 Nm, suggested its possible involvement in providing mobility support, enabling swift kicking actions and defensive blocks. Lastly, the hip joint showed a moment of −27.2661 Nm, indicating a potential role in coordinating body movements and in assisting force distribution during combat (Figure 10).

### Kick Posture

The supporting leg’s role seemed to absorb the impact and provide the necessary stability and counterforce for the kick. The forces at the joints of the supporting leg, including the ankle, probably suggest coordinated action across the leg to maintain posture and balance during the kick.

The moments demonstrated a potentially stabilizing function and the leg’s contribution to resisting the kick’s force and maintaining the fighter’s balance. The knee joint showed the highest moment at 64.2947 Nm, along with the hip joint at −32.8831 Nm. The kicking leg exhibited markedly lower moments, with a nearly negligible moment at the ankle 0.033354 Nm and modest moments at the knee and hip.

## 4. Discussion

The exploration into the biomechanical characteristics of ancient athletics, specifically through the lens of the depicted postures and movements as exhibited in the practices of ancient Greek sports, may yield insights that can be characterized as both novel and enlightening.

This study, to the best of our knowledge, represents a pioneering effort in the static biomechanical analysis of ancient athletes’ postures using modern computational tools such as computer vision and static multi-segmental analysis.

### 4.1. Considerations of the Examined Postures Weight Distribution and Ground Reaction Forces

The first parameter examined in this research was the weight distribution of the athlete’s body weight at the initial phase of a match. Regarding the weight distribution of an athlete’s body during the initial phase of the engagement, this analysis estimated an allocation of body mass, with a posterior distribution with 68% of the weight positioned on the hind leg. This could be interpreted as a biomechanical adaptation designed to optimize performance. This disparity in vertical force application between the posterior and anterior extremities may represent a sophisticated approach to leveraging body mechanics for a competitive advantage. The hind leg emerged as a critical element for stability and power generation [40,41,42]. This suggests that the hind leg’s role diverged from modern combat methodologies. Whereas the hind leg appeared to serve as the primary stability anchor in ancient Pankration, modern striking disciplines demonstrate alternate biomechanical patterns [43]. Unlike the rotational and weight-transfer techniques common in modern disciplines [44], Pankration may have predominantly relied on a more fixed weight distribution on the hind leg.

On the other hand, the front leg carried a significantly smaller load, suggesting a role in maneuverability, and held the potential to contribute to balance during dynamic movements. In martial arts like Karate and Muay Thai, the dynamic transfer of weight to the front leg allows for rapid adjustments in stance [45]. Similarly, Muay Thai uses a lighter front leg to enable quick shifts in stance and balance adjustments during strikes [46]. In contrast, Pankration’s weight distribution prioritized stability by minimizing weight on the front leg, a vulnerable target for opponents [24]. This may have been due to the high risk of unbalanced kicks, which could lead to significant disadvantages in a sport that heavily relied on grappling and throwing techniques, as illustrated in Figure 2. The front leg’s reduced loading would possibly facilitate quicker more agile adjustments in position. It should be noted that this imbalance in force distribution seems to indicate an advanced understanding of body mechanics, considering it was developed more than 2500 years ago. This understanding leveraged the limbs for specific functional roles: the hind leg for power and stability and the front leg for agility and balance.

The analysis of horizontal ground reaction forces (GRFs) could potentially offer further insight into the dynamics of movement too. Knowing that the matches used to take place in a sanded arena (palestra), the hind leg signified the force exerted backward against the ground, which, in turn, propels the body forward [31,47,48]. As the horizontal forces are also used in modern combat sports for initiating movement and producing momentum, we are able to understand one of the ancient approaches in power generation [49].

Additionally, it seemed that the strategic postural manipulation might have enabled the athletes to selectively engage targeted muscle groups, resulting in a biomechanically efficient system for posture maintenance. A feature we observed in our simulation is the high-magnitude forces and moments while in static equilibrium, a phenomenon derived by bodily configuration, specifically the articular angles formed at each joint throughout the kinematic chain. This is characterized as a fundamental component of effective posture manipulation [50]. By knowing that the passive elastic elements in the leg’s muscles store potential energy, which is then converted into kinetic energy to initiate motion, it is possible that the athlete used this as a sophisticated method of storing potential energy while activating targeted muscles [42].

Regarding the kick posture, the full GRF is exerted on the base leg, since it is the only one that is in contact with the ground. Here, it is imperative to acknowledge the multifaceted functionality of the lower extremity, which encompasses attenuation of impact forces, alongside the provision of stability and propulsion. The analysis of the horizontal GRF elucidates the requisite lateral stabilization for equilibrium maintenance. The elevated mechanical outputs of the base leg may have contributed substantially to the reduction in kinetic energy during impact, which mitigates the risk of losing balance and facilitates strategic positioning and movement [51]. The suggested robust activation of the muscles is essential for pelvic stabilization and limb control at the final phase of the kick and significantly enhances the biomechanical characteristics of the kick [52]. Differences were revealed compared to modern martial arts. The weight distribution in Pankration shows that most of the body weight was placed on the hind leg even in the readiness posture, which suggests a stance that contrasts with the more dynamic weight distribution seen in modern combat sports like Karate and Taekwondo [45,53]. The distribution of body weight in modern sports facilitates quicker transitions between offensive and defensive movements. Another significant difference lies in rotational movements: modern kicking techniques, such as the roundhouse kick, involve substantial hip rotation and pivoting on the supporting foot to generate speed and force [54]. In contrast, the Pankration stance indicates a possible tendency to a more linear approach, which may reduce kick speed and range. Unlike martial arts, where athletes frequently utilize pulsing movements, small jumps, and rapid shifts in weight to generate momentum and amplify strike force [55], the Pankration posture analyzed here presented a well-grounded stance with a low CoM and high joint moments produced during the posture. These differences suggest that ancient Pankration athletes may have employed alternative methods of power generation, focusing on stability and controlled force transfer rather than the explosive momentum-driven techniques seen in modern practices.

In terms of stance configurations, modern martial arts prioritize stances that enable rapid direction changes and swift execution of strike combinations [56]. The ancient Pankration stance, while offering stability and strength, likely reflects different combat objectives, favoring prolonged stability over agility, possibly due to different rules or the absence of modern time constraints. From an optimal perspective, the Pankration stance can be seen as contextually effective for its historical setting. The static stable posture likely suited the conditions of ancient combat, which may have involved different opponent tactics and lacked protective gear, necessitating stronger more grounded postures. However, in terms of biomechanical efficiency, this stability likely sacrificed some agility and speed attributes that modern martial arts balance through more versatile rotational stances [57].

The evolution of techniques in combat sports has been driven by advances in biomechanics and sports science. While ancient Pankration postures were biomechanically effective within their context, modern techniques have optimized these foundations, balancing power with speed and flexibility.

### 4.2. Strategic and Training Implications of the Analysis in Athletic Performance

The static analysis revealed potential insights into athletes’ tactical body positioning strategies too [58,59]. The distribution of the moments was possibly designed to maximize both stability and power generation, considering the existing literature. The augmentation of joint moments beyond typical anatomical norms could introduce unpredictability and athlete’s movements, obscuring their tactical intentions and potentially deterring opponent aggression [60,61]. The analyzed postures might have allowed athletes to effectively control the pace of engagement, influence their opponent’s decision-making, and execute potent counterattacks. As mentioned by Theocritus, Amycus in his fight attempted to position his body to gain the advantage of sunlight and use it against his opponent [59]. Additionally, the necessary muscle activation needed to counteract the moments could help athletes, providing them with the capability to react to opponents’ moves, thus maintaining a versatile offensive and defensive strategy [62].

The estimated static postural loads establish a baseline for developing the benefits developed through isometric training, i.e., tendon stiffness, joint stability, and neuromuscular control [63]. Sustained isometric tensions enhance muscle activation and joint integrity, facilitating the body’s methodical priming for maximal force production [64]. These findings align with ancient practices noted by Galen, where static exercises were used to enhance muscular endurance and strength. Isometric holds, characterized by continuous motor unit activation, promote intramuscular coordination and muscle fiber recruitment, contributing to muscular endurance and hypertrophy [65,66]. According to Galen, combining static and dynamic exercises within training programs provided balanced physical conditioning, with static postures offering foundational stability and endurance necessary for dynamic power [65,67]. These findings suggest that ancient athletes possibly optimized their stances for stability and agility, employing biomechanical principles that remain relevant in modern athletic training.

Summarily, the findings suggest that ancient athletes optimized their stances for both stability and agility, using biomechanical principles that align with modern theories a feature not only documented by our findings but also described in the ancient texts [24,37]. The readiness posture’s weight distribution supports the development of tendon stiffness and neuromuscular control, crucial for all combat sports [68,69]. It is speculated that the strategic use of dorsiflexion in the kick posture emphasizes force concentration, maximizing impact and minimizing energy loss. Overall, the static analysis constitutes a preliminary step toward the exploration of the technical intricacies of ancient combat sports. Simultaneously, it establishes a framework for the reconstruction of Pankration closer to its original form.

### 4.3. Limitations

The research on biomechanical insights and strategic applications in ancient combat sports, while pioneering, is subject to several limitations. Reliance on artistic pottery depictions may introduce inaccuracies, as these representations might not accurately capture the dynamic and realistic postures of actual combatants. Conducting a two-dimensional analysis of inherently three-dimensional movements limits the comprehensiveness of force and moment estimations, potentially leading to incomplete biomechanical insights. Assumptions made in calculating the CoM based on a hypothetical athlete model may not account for the diverse body types of ancient athletes, which affected the accuracy of the joint force and moment calculations. Additionally, the exclusive focus on static postures does not encompass the dynamic nature of combat sports and possibly leads to overlooking biomechanical strategies employed during movement. Lastly, the use of OpenPose on stylized historical artwork may result in keypoint detection errors, undermining the reliability of the biomechanical models and the study’s overall validity. The impact of relying on artistic depictions and a hypothetical model on result validity needs to be further explored.

## 5. Conclusions

The present research into the biomechanical characteristics of ancient Greek athletics was conducted using computer vision and static multi-segmental analysis of the lower extremities. The analysis demonstrated weight distributions and effective utilization of GRFs, which aligned with ancient accounts of strategic positioning to enhance the technique in both offense and defense [24,59]. It was found that, while modern front kick techniques prioritize velocity and dynamic force, the ancient Pankration front kick based its effectiveness on stability and controlled force application, which is justified by the high magnitude of joint moments in the base leg. Meanwhile, sustained muscle tension during the analyzed postures was likely used to counterbalance the forces and moments produced by the estimated configurations. According to the literature, this was possibly used to improve coordination and muscle fiber recruitment, as mentioned by the historical evidence, where isometric contractions were used to enhance muscular endurance [24]. The study also provided insights into how equilibrium was attained, which are elements that contribute to overall athletic performance.

The research also argued that isometric training could be used to improve neuromuscular control, tendon stiffness, and joint stability, features that have practical applications in modern sports science. The examination of weight distribution and GRFs offered insights for optimizing athletic training and performance. Finally, the use of advanced computational tools presented a methodological advancement in the biomechanical analysis of both historical and modern athletic practices.

## 6. Practical Applications

This study offers practical applications for combat sports athletes and coaches leveraging insights from ancient Greek athletic practices. The main application is the use of static analysis to improve fighting stances and techniques. Unlike their extensive use in traditional Asian martial arts, isometric exercises and static postures are less common in modern combat sports [70,71]. However, the study emphasized that they should be more widely employed to enhance joint stability and tendon stiffness, as they were in antiquity. Incorporating static postures from Pankration into modern training may improve athletes’ proprioception and balance. By practicing stances that require maintaining stability under load, athletes can develop stronger stabilizer muscles and improve joint integrity. Also, understanding the effectiveness of static postures in Pankration can offer tactical advantages in modern combat sports. Athletes can incorporate elements of surprise and control by mastering techniques that rely on sudden force generation from stable positions, rather than constant movement in the field. Coaches and athletes might experiment with integrating static power generation methods from Pankration into modern training. Additionally, the software used in the research methodology can be employed to provide feedback on postural analysis while it offers a wide range of applications in the field of sports science. Finally, the study’s results can foster a deeper appreciation for cultural heritage, leading to the revival of ancient sports in a modern context.

### Future Research

Future studies should build upon the foundational insights provided by this investigation, focusing on several key areas to further elucidate the biomechanics of ancient athletic practices. Future research should also focus on integrating the biomechanical principles of ancient practices into modern athletic training and aim through the ancient practices to enhance performance, reduce injury risk, and improve rehabilitation.

First, there is a need to extend the analysis to dynamic movements, incorporating motion capture and advanced computational models to simulate the full range of athletic maneuvers performed in ancient sports such as Pankration. This would provide a more comprehensive understanding of the forces, moments, and muscle activations involved in both offensive and defensive actions. Additionally, future research should investigate the interplay between static and dynamic postures to assess how transitions between these states contribute to athletic performance and injury prevention.

This includes examining the role of isometric exercises in developing muscle strength and joint stability, as well as their impact on dynamic activities. In particular, isometric training techniques, as reflected in ancient static postures, could be explored for developing muscle strength, joint stability, and neuromuscular control, which is not currently happening. Another important area for future exploration is the application of modern biomechanical analysis tools to a broader range of historical athletic practices and artifacts. Importantly, future studies should focus on reevaluating ancient knowledge, especially for sports with origins in the ancient world, ensuring that modern optimizations are grounded in historically informed biomechanics. This approach shifts the focus from purely historical simulations to practical interdisciplinary innovations, ensuring that ancient biomechanical principles continue to offer value in modern sports science and biomechanics. Finally, because ancient sports were developed under distinct sociocultural conditions, they may possess intricate features that remain undiscovered. The concept of sports in antiquity was predominantly shaped by a warfare-oriented approach, potentially influencing the kinesiology of athletes. The investigation into these historical practices, which were developed through a lens markedly different from contemporary perspectives, may reveal novel aspects of athletic performance and development.

## Figures and Tables

**Table 1:** Segmental dimensions used in the present simulation.

Segment	Length (cm)
Foot	24
Lower Leg	48
Upper Leg	50

**Table 2:** Normative data on the relative mass and the location of the Centre of Mass for each anatomical segment.

Segment	Relative Mass (%)	Centre of Mass (% from Proximal End)
Upper Leg	10.7	43.9
Lower Leg	4.7	42.0
Foot	1.7	43.4

## Data Availability

Not applicable.
